# Potential Association of Legionnaires’ Disease with Hot Spring Water, Hot Springs National Park and Hot Springs, Arkansas, USA, 2018–2019

**DOI:** 10.3201/eid2801.211090

**Published:** 2022-01

**Authors:** Allison E. James, Kurt Kesteloot, J. Terry Paul, Richard L. McMullen, Shirley Louie, Catherine Waters, Jennifer Dillaha, Joel Tumlison, Dirk T. Haselow, Jessica C. Smith, Sooji Lee, Troy Ritter, Claressa Lucas, Jasen Kunz, Laura A. Miller, Maria Said

**Affiliations:** Centers for Disease Control and Prevention, Atlanta, Georgia, USA (A.E. James, J.C. Smith, S. Lee, T. Ritter, C. Lucas, J. Kunz);; US Public Health Service, Rockville, Maryland, USA (K. Kesteloot, T. Ritter, M. Said);; US National Park Service, Washington, DC, USA (K. Kesteloot, L.A. Miller, M. Said);; Arkansas Department of Health, Little Rock, Arkansas, USA (J.T. Paul, R.L. McMullen, S. Louie, C. Waters, J. Dillaha, J. Tumlison, D.T. Haselow)

**Keywords:** Legionnaires’ disease, Legionella, environmental health, Legionellosis, hot springs, Hot Springs National Park, Arkansas, United States, bacteria

## Abstract

*Legionella pneumophila* is the cause of Legionnaires’ disease, a life-threatening pneumonia that occurs after inhalation of aerosolized water containing the bacteria. *Legionella* growth occurs in stagnant, warm-to-hot water (77°F–113°F) that is inadequately disinfected. Piped hot spring water in Hot Springs National Park, Arkansas, USA, has naturally high temperatures (>135°F) that prevent *Legionella* growth, and Legionnaires’ disease has not previously been associated with the park or other hot springs in the United States. During 2018–2019, Legionnaires’ disease occurred in 5 persons after they visited the park; 3 of these persons were potentially exposed in spa facilities that used untreated hot spring water. Environmental testing revealed *Legionella* bacteria in piped spring water, including 134°F stagnant pipe water. These findings underscore the importance of water management programs to reduce *Legionella* growth in plumbing through control activities such as maintaining hot water temperatures, reducing stored water age, and ensuring adequate water flow.

Legionnaires’ disease is caused by inhalation of water containing *Legionella* bacteria and can lead to life-threatening pneumonia. *Legionella pneumophila* bacteria thrive in water temperatures of 77°F–113°F, and exposure commonly occurs by inhaling contaminated warm water that is aerosolized, such as during bathing in whirlpool tubs or showering (*1*,*2*). In buildings, stagnant water; presence of scale, sediment, or biofilms in pipes; inadequate levels of disinfectant; pH fluctuations; and favorable water temperatures in the building’s plumbing are known risks for *Legionella* growth (*1*).

Approximately 1.5 million persons visit Hot Springs National Park (HSNP), Arkansas, USA, each year. The major attraction to HSNP is the row of 8 bathhouses built during 1892–1923 (*3*). Only 2 bathhouses continue to operate as spas, providing beauty and health treatments using untreated hot water piped into the buildings from nearby springs. At >135°F, the natural temperature of HSNP hot spring water is unfavorable for *Legionella* growth and exceeds the recommendation by the National Academies of Science, Engineering, and Medicine (NASEM) to maintain water temperatures >131°F to prevent *Legionella* growth. However, a heat exchange system in HSNP cools some of the untreated hot spring water to 75°F–95°F for use in bathhouses to avoid scalding visitors. Despite the cooled water being in temperature range of optimal *Legionella* growth, Legionnaires’ disease has not previously been associated with visiting HSNP or other hot springs in the United States. *Legionella* spp. have been found in untreated hot spring water in other countries and have been reported as the source for Legionnaires’ disease in outbreaks (*4*–*6*).

Because Legionnaires’ disease is a nationally notifiable condition, cases are systematically reported to the Centers for Disease Control and Prevention (CDC) by state and local health jurisdictions. Potential exposures for persons meeting the Legionnaires’ disease surveillance case definition who spend any nights away from home in the 10 days before symptom onset are reported to the jurisdiction where the exposure might have occurred through CDC’s Supplemental Legionnaires’ Disease Surveillance System and travel-associated case notification reports. During July 2018–October 2019, the Arkansas Department of Health (ADH) and National Park Service Office of Public Health (NPS-OPH) were notified of 5 persons in whom Legionnaires’ disease occurred after traveling to HSNP and Hot Springs, Arkansas. During August 2018–October 2019, the 2 organizations jointly investigated patients’ exposures to water while traveling to identify potential sources of *Legionella* bacteria and recommend mitigation strategies.

## Methods

### Case Finding, Patient Interviews, and Exposure Assessment

CDC *Legionella* travel-associated case notification reports included name and address of travel accommodations, potential exposures to aerosolized water or spas, test used for diagnosis, and whether the patient survived. Cases were defined as illness in patients who met the surveillance case definition and experienced symptoms of Legionnaires’ disease within 10 days after visiting HSNP. When additional exposure information was needed, local health jurisdictions where the patient lived shared case interview forms with ADH and NPS-OPH (3 patients) or patients or next-of-kin were reinterviewed by ADH (2 patients). Additional case finding was attempted through Epidemic Information Exchange (CDC, https://emergency.cdc.gov/epix) notifications (sent July 11, August 5, and October 5, 2019), bathhouse guest notifications, and press releases. Patient data were saved on secure NPS-OPH and ADH network servers, and relevant *Legionella* exposure information obtained from patients or next-of-kin was entered into an Excel (Microsoft, https://www.microsoft.com) spreadsheet and shared between NPS-OPH and ADH by using the US National Park Service (NPS) secure file transfer service.

### Environmental Assessment and *Legionella* Testing

An ADH environmental health specialist evaluated all accommodations and places visited in Hot Springs, Arkansas, listed by patients for *Legionella* risk. ADH and NPS-OPH staff inspected the HSNP hot spring water distribution system, including plumbing, reservoirs, and point-of-use sites in bathhouses and fountains. Environmental samples were collected for *Legionella* culture testing from the bathhouses, reservoirs, jug-filling stations, decorative fountains, or cooling towers on 6 occasions during October 2018–November 2019. Sampling sites during each round of testing were selected on the basis of epidemiologic information at the time and results of previous environmental tests. Laboratory A or laboratory B performed the testing; both laboratories were members of the Environmental *Legionella* Isolation Techniques Evaluation Program.

### Ethics 

This investigation was completed as part of routine outbreak response activities by ADH and NPS-OPH. These activities were further reviewed by CDC and determined to be not research.

## Results

### Water System Description

In HSNP, 2 bathhouses perform spa services with spring water (bathhouses A and B). An additional bathhouse was converted into a hotel that pipes spring water to guest rooms for baths (bathhouse C). Of the remaining 5 bathhouses, 1 is vacant, and the others house HSNP staff offices and a retail store (bathhouse D), a brewery restaurant (bathhouse E), a visitor center, and a cultural center. Except for the visitor center, all businesses operate as private entities and maintain a contract with HSNP to operate in park facilities. A hot spring water reservoir is located under a park administration building, which is also on bathhouse row (Figure). A hotel spa facility (facility X) and a medical center (facility Y) adjacent to HSNP also offer beauty or health treatments using hot spring water piped from the HSNP. A total of 3 hot water jug-filling fountains, 2 within HSNP and 1 outside the park in Hot Springs, enable visitors and residents to fill containers with hot spring water that meets US Environmental Protection Agency drinking water requirements because of the disinfectant properties of naturally high water temperatures. In addition, 4 decorative hot spring water fountains and 2 natural display hot water springs are inside park boundaries.

**Figure:**
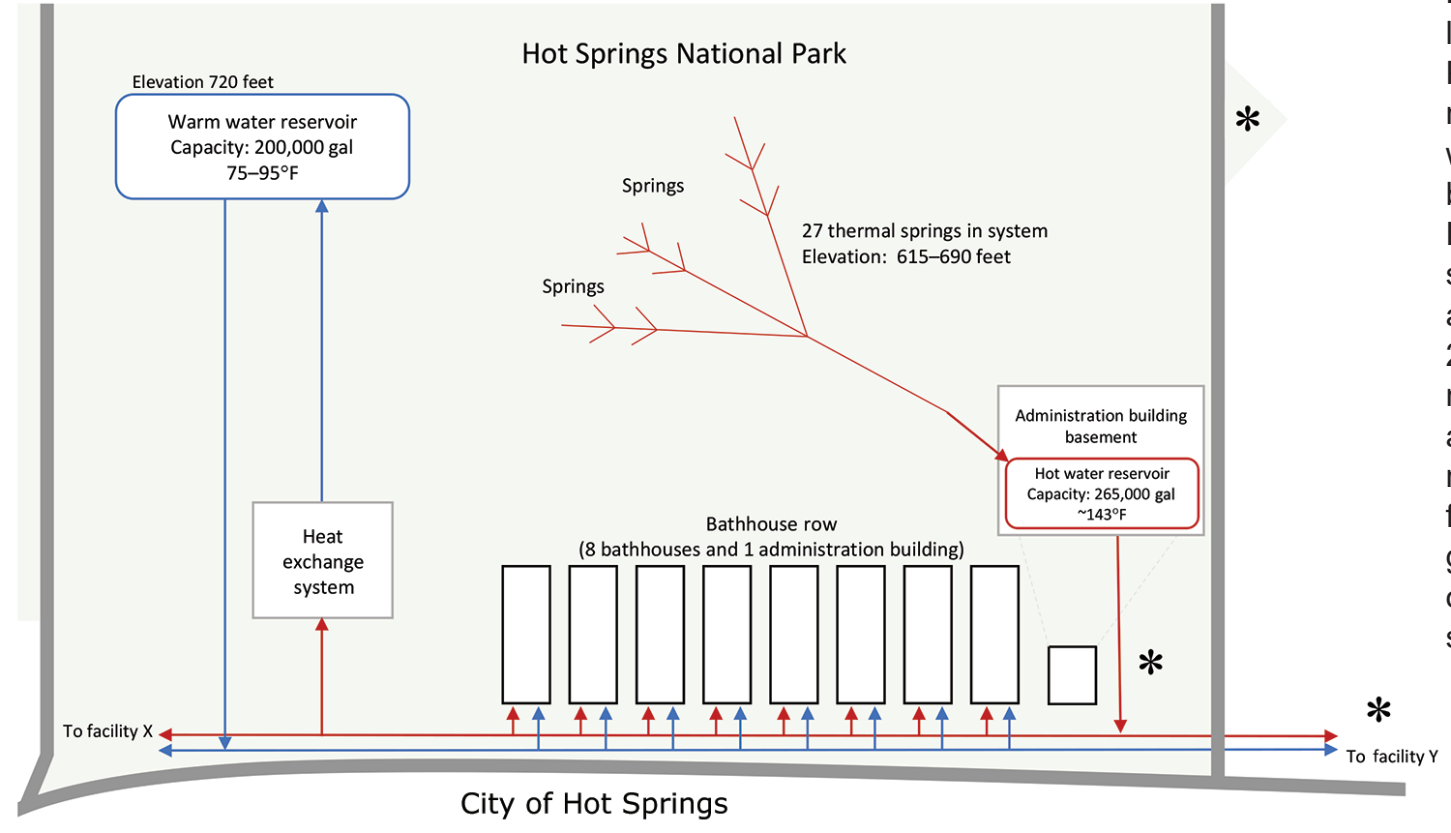
Simplified flowchart and location of Hot Springs National Park water distribution system relative to bathhouses, hot spring water jug-filling stations (depicted by asterisks), and the city of Hot Springs, Arkansas, USA, in study of Legionnaires’ disease association with hot springs, 2018–2019. Accommodation Z is not plumbed to hot spring water and is not shown. Warm water reservoir capacity was halved, from 400,000 gallons to 200,000 gallons, in response to detection of *Legionella* spp. in piped hot spring water.

All 27 hot springs that supply HSNP are covered, sealed, and protected, and hot spring water is collected in a complex water distribution system (Figure). First, the hot spring water is piped into an ≈265,000 gallon reservoir. From the reservoir, the water is pumped to the bathhouses, an additional 100,000 gallon reservoir (not depicted in Figure), and a heat exchange system that cools the >135°F water to 75°F–95°F. This warm water is then pumped to a 400,000-gallon reservoir (later modified to 200,000-gallon capacity after the outbreak). Hot and warm water reservoirs are at a higher elevation than the bathhouses and provide constant water pressure. Bathhouse spring water temperature is adjusted by mixing warm and hot water, but water temperature cannot exceed 104°F in full body–contact recreational water (such as hot tubs or pools), according to NPS regulations (*7*). NPS regulations also stipulate that cooled hot spring water used in pools or for purposes more likely to result in inhalation of water droplets, such as individual-use jetted tubs, must be chemically disinfected (e.g., treated with chlorine or ozone).

### Patient Demographics, Potential Exposures, and Diagnostic Testing

Case finding revealed no additional cases beyond the 5 patients reported by CDC who met the case definition. Symptom onset occurred during July 2018–August 2019 for the 5 travelers who received a Legionnaires’ disease diagnosis (Table 1). Patient age range at diagnosis was 61–72 years. Among the 4 patients who survived, all had underlying health or behavioral risk factors for Legionnaires’ disease, including chronic obstructive pulmonary disease, smoking, or daily use of a continuous positive airway pressure therapy machine. Next-of-kin reported no underlying health or behavioral risk factors for the patient who died.

**Table 1:** Legionnaires’ disease patients’ age, illness onset date, type of diagnostic test, and shared and probable exposures while traveling in HSNP and Hot Springs, Arkansas, USA, 2018–2019*

Patient	Patient age, y†	Illness onset date	Confirmatory diagnostic test	Shared exposures‡	Probable exposure§‡
1	65	2018 Jul 27	Sputum PCR	HSNP, bathhouse A¶	Bathhouse A
2	72	2018 Nov 23	Urinary antigen	HSNP, bathhouse A, accommodation Z	Bathhouse A
3	67	2019 May 7	Urinary antigen	HSNP, accommodation Z	Unknown
4#	61	2019 Jun 22	Sputum PCR	HSNP, bathhouse A	Bathhouse A
5	66	2019 Aug 25	Urinary antigen	HSNP	Unknown

*HSNP, Hot Springs National Park. †Age in years at the time of illness onset. ‡Only potential exposures shared with >1 other patients are listed. §Based on epidemiologic investigation and environmental test results. ¶Exposures at bathhouse A might have included swimming, bathing, or showering in untreated hot springs water. #Deceased; patient received negative urinary antigen test result.

Among the 5 patients with Legionnaires’ disease, all had visited HSNP; 2 stayed >1 nights at accommodation Z near HSNP (patients 2 and 3, not in the same room), and 3 patients received hot spring water spa services from bathhouse A (patients 1, 2, and 4). One patient (patient 5) did not enter bathhouse A or stay in accommodation Z. A common likely exposure source for the 2 patients who did not visit bathhouse A (patients 3 and 5) was not identified.

In 2 patients, Legionnaires’ disease was diagnosed by a PCR assay on lower respiratory specimens with no serogroup reported (patients 1 and 4); in 3 other patients the diagnosis was made by urinary antigen test, suggesting infection with *Legionella pneumophila* serogroup 1 (patients 2, 3, and 5) (*8*). Specimens from 1 person (patient 4) underwent both urinary antigen and sputum PCR testing, but only the sputum PCR test was positive. No patient specimens were submitted for culture testing, because samples were discarded by the time cases were reported to ADH and NPS-OPH.

### Environmental Assessment and Testing

An environmental inspection of accommodation Z after the patients’ visits revealed that the building was plumbed to municipal water only, and faucet water within guest rooms was found to contain residual chlorine disinfectant at hot and cold temperatures. No specific risks for *Legionella* growth were identified in accommodation Z, and water was not submitted for *Legionella* culture testing. ADH and NPS-OPH inspected bathhouse A after each of the first 2 patients who visited the spa facility were reported. At that time, no specific risks for *Legionella* growth were identified. All faucets plumbed to municipal water contained residual chlorine disinfectant, and all faucets plumbed to spring water could reach temperatures >135°F. After receiving the travel-associated case notification report of the third patient who visited bathhouse A, an August 2019 inspection by ADH discovered that water ozonators intended for water disinfection on the individual-use microbubbling jetted soaking tubs were detached. The length of time ozonators had been detached is unknown. A final inspection in September 2019, prompted by a positive *Legionella* test from a poolside shower, found the shower to be plumbed to cooled hot spring water rather than municipal water.

All environmental samples collected during October 2018, January 2019, and July 2019, after each of the first 3 Legionnaires’ disease cases were reported, were sent to laboratory A and tested negative by culture for *Legionella* (Table 2). On September 18, 2019, additional environmental samples were collected after the fourth patient was reported (the third with a potential exposure in bathhouse A). This round of testing was the first to use laboratory B and the first to produce a positive result; the positive sample was collected from a hot spring water poolside shower in bathhouse A and was determined to be *L. pneumophila* serogroup 2–14 (Table 3). The positive *Legionella* culture prompted extensive testing throughout HSNP in October 2019, revealing *L. pneumophila* serogroup 2–14 in hot springs water in 1 additional site in bathhouse A, hot and cooled spring water from an infrequently used faucet in bathhouse C, and a hot spring water spigot in the spa area of facility X. Spring water temperatures were recorded only during the October and November 2019 round of testing. The positive sample from bathhouse A was taken from a hot water (134°F) pipe with stagnant or low water flow in the basement (Table 3). Additional parkwide testing in November 2019 produced 1 positive sample from the cooled spring water supply pipe in facility Y. Facility Y staff indicated that the cooled spring water supply line is infrequently used and is not routinely flushed. All samples from the HSNP hot and warm water reservoirs, main hot spring water line, and heat exchange system tested negative for *Legionella*. None of the *L. pneumophila* from HSNP’s water samples that tested positive belonged to serogroup 1.

**Table 2:** Environmental sample collection dates and locations, laboratory, and *Legionella* spp. culture results, HSNP and Hot Springs, Arkansas, USA, 2018–2019*

Sample collection date	Laboratory	Locations†	Culture result
2018 Oct 3	A	Bathhouse A (poolside shower, individual shower, nearby cooling tower)	Negative
2019 Jan 9	A	Bathhouse A (poolside shower, waterfall in 102°F pool, shower in women's locker room, shower in men's locker room, sauna cave, soaking tub, nearby outdoor cooling tower)	Negative
2019 Jul 15–16	A	Bathhouse A (pool area air-conditioning unit, basement wood board, pool filter, hot water main supply line)	Negative
		5 decorative outdoor fountains	Negative
		Administration building hot water reservoir	Negative
		2 nearby cooling towers	Negative
2019 Sep 18	B	Bathhouse A (**poolside shower**)‡	Positive
		2 decorative fountains near HSNP boundary	Negative
		Administration building hot water reservoir	Negative
2019 Oct 4	B	Bathhouse A (soaking tub, **basement pipe**)	Positive
		Bathhouse B (soaking tub)	Negative
		Bathhouse C (**hot and cooled water soaking tub faucets**)	Positive
		Bathhouse D (sink)	Negative
		Bathhouse E (sink)	Negative
		Hot springs waterfall display	Negative
		Hot springs water jug-filling station	Negative
		Cooled or tempered water reservoir	Negative
		4 decorative outdoor fountains	Negative
		Facility X (**hot spring water spigot**)	Positive
		Nearby cooling tower	Negative
2019 Nov 12	B	Administration building (hot spring water reservoir, 2 pump valves, hot water overflow)	Negative
		3 jug fountains	Negative
		1 decorative outdoor fountain	Negative
		Bathhouse A (basement valve)	Negative
		Bathhouse B (basement valve)	Negative
		Bathhouse C (basement valve, 2 guest room soaking tubs)	Negative
		Bathhouse E (basement valve)	Negative
		Facility X (2 pump valves, 2 basement valves)	Negative
		Hot springs water reservoirs (4 samples)	Negative
		Facility Y (hot spring water supply, **cooled spring water supply**)	Positive

*Bold text denotes locations of positive samples. HSNP, Hot Springs National Park. †Bathhouses A and B are spas; bathhouse C is a hotel; bathhouse D houses staff offices and a retail store; bathhouse E is a brewery restaurant. Facility X is a hotel and spa; facility Y is medical center.

**Table 3:** Positive environmental culture sample collection dates and locations, *Legionella* species and serogroup, and temperature of sampled water, Hot Springs National Park and Hot Springs, Arkansas, USA, 2018–2019

Sample collection date	Location of positive sample	Species/serogroup	Temperature of sampled water*
2019 Sep 18	Bathhouse A (poolside shower)	*Legionella pneumophila* 2–14	Not documented
2019 Oct 4	Bathhouse A (basement pipe)	*L. pneumophila* 2–14	134°F
	Bathhouse C (hot water soaking tub faucet)	*L. pneumophila* 2–14	128°F
	Bathhouse C (cooled water soaking tub faucet)	*Legionella* spp.	96°F
	Facility X (hot springs water spigot)	*L. pneumophila* 2–14	119°F
2019 Nov 12	Facility Y (cooled spring water supply)	*L. pneumophila* 2–14	73°F

*Water temperatures were not documented until the October 2019 round of testing.

## Discussion

For 3 of 5 patients, this investigation suggested that the potential source of *Legionella* exposure was untreated hot springs water used during spa services in bathhouse A. This notion is supported by environmental testing that revealed the presence of *Legionella* in hot spring water in bathhouse A, where patients reported being in or near aerosolized water during their exposure period. No other likely exposures outside of bathhouse A were identified for these 3 patients. The exposure for the 2 patients who did not visit bathhouse A is less clear, but both reported spending time in HSNP near the bathhouses. Although untreated warm water is known to be conducive to *Legionella* spp. growth, hot spring water has not previously been associated with Legionnaires’ disease in the United States.

Environmental testing during this investigation revealed viable *Legionella* bacteria in 134°F stagnant pipe water, exceeding both the optimal growth range of 77°F–113°F for *Legionella* and the NASEM recommended water maintenance temperature of 131°F for the prevention of *Legionella* growth (*1*,*9*). These findings indicate that potential for *Legionella* growth from very hot spring water exists. However, despite multiple reports of environmental sampling confirming the presence of *Legionella* spp. in recreational hot springs water, outbreaks are rarely reported (*4**–**6*). Why Legionnaires’ disease cases associated with HSNP and bathhouse A were detected beginning in 2018 is unclear. No changes to the hot springs water distribution system or bathhouse A were reported before the index patient was reported. In recent years, reporting to CDC’s Supplemental Legionnaires’ Disease Surveillance System has increased, resulting in improved coordination and timely reporting of out-of-state legionellosis cases to public health jurisdictions where a potential exposure occurred, which might have contributed to detection of this cluster.

As a result of this investigation, NPS-OPH and ADH recommended a comprehensive water management program for HSNP, businesses within HSNP, and businesses using HSNP spring water outside of park boundaries. All entities using HSNP hot springs water voluntarily adopted water management programs to mitigate *Legionella* growth in piped, untreated hot spring water (*10*). Minimum program requirements were that water likely to be inhaled (e.g., showers) was plumbed to city potable water, quarterly parkwide *Legionella* culture testing would continue until results were negative for >2 consecutive test cycles, and water to individual tubs that provide microbubbles or jets must be chemically disinfected. Daily flushing of hot spring water pipes in each building until faucet temperatures reached >135°F was also required. This flushing temperature requirement exceeds the >131°F recommended by the NASEM, but because HSNP spring water is naturally hotter (>140°F), 135°F was chosen to indicate water turnover in building plumbing (*9*). The plan also included stipulations for chemical and manual cleaning of tubs and steam showers to minimize biofilm formation, a recommendation to provide a visible notice to spa patrons about *Legionella* risk from aerosolized untreated hot spring water, and a requirement for spa businesses to maintain logs of customer contact information for risk notification if water testing reveals presence of *Legionella.* Finally, a structural modification was made to the warm water reservoir, reducing its capacity by half, from 400,000 gallons to 200,000 gallons, to increase water turnover and potentially reduce biofilm formation. HSNP is responsible for ensuring continuity of the water management program with NPS-OPH oversight.

The water management program was finalized and put in place in January 2020. Because of park closures and operations modifications in response to the coronavirus disease pandemic beginning in March 2020, the quarterly water testing plan was interrupted. As of December 2021, no new cases meeting the outbreak case definition have been reported to ADH or NPS-OPH. However, park and bathhouse visitation decreased during the pandemic, which might have resulted in fewer potential exposures.

Despite epidemiologic evidence that implicates bathhouse A as the source for *Legionella* exposure in 3 patients, the link is not definitive because of 3 study limitations. First, patient test results lacked complete *Legionella* species and serogroup information, and no isolates were available for whole-genome sequencing. The lack of more complete laboratory data hampered the ability to genetically compare isolates from potential environmental exposure sources with patient specimens. Moreover, 3 of 5 patients tested positive by the urinary antigen test (suggestive of infection with *L. pneumophila* serogroup 1), but none of the environmental samples were culture-positive for *L. pneumophila* serogroup 1. However, the urinary antigen test has been shown to cross-react with *L. pneumophila* serogroups 2–14 and other *Legionella* species (*11*). The second limitation is the low sensitivity of environmental cultures for *Legionella*, which might have impeded detection of the bacteria from more sites. These bacteria are inherently difficult to culture from environmental water samples because *Legionella* is slow-growing and has unique nutritional requirements (*12*). *Legionella* bacteria are also intermittently released from biofilms in plumbing, which might result in negative culture results at the time of sample collection (*13*). Whether the second laboratory that reported the only positive results used more sensitive culture methods than the first, or if earlier samples were truly negative, remains unknown. Third, case report forms were not standardized across public health jurisdictions, and case interview forms were unlikely to capture all possible exposures. Relatedly, 2 of 5 patients could not be epidemiologically linked to a specific likely exposure, except that they had visited HSNP during their exposure period, raising the possibility that another unrecognized common exposure existed.

This investigation identified untreated hot spring water as a potential source for Legionnaires’ disease. In facilities where untreated hot spring water is collected for distribution in plumbing and used for activities that might result in water aerosolization (e.g., showering or whirlpool tub bathing), adopting water management programs can reduce the risk for *Legionella* growth (*14*). Effective programs might include routinely flushing plumbing until a minimum temperature of >131°F is achieved at the faucet, limiting use of untreated water in showers, and making structural modifications that reduce system water age (*9*). Additional aspects of effective programs incorporate inspection of water distribution systems and eliminating pipes with stagnant water. Finally, healthcare providers should consider Legionnaires’ disease among differential diagnoses for patients with pneumonia and a history of recent exposure to hot spring water.
